# Draft genome of *Myxosarcina sp.* strain GI1, a baeocytous cyanobacterium associated with the marine sponge *Terpios hoshinota*

**DOI:** 10.1186/s40793-015-0011-3

**Published:** 2015-05-27

**Authors:** Chun-Hui Yu, Chung-Kuang Lu, Huang-Ming Su, Tzen-Yuh Chiang, Chi-Chuan Hwang, Tsunglin Liu, Yi-Min Chen

**Affiliations:** 1 Institute of Bioinformatics and Biosignal Transduction, National Cheng Kung University, Tainan 701, Taiwan; 2 Department of Engineering Science, National Cheng Kung University, Tainan 701, Taiwan; 3 National Research Institute of Chinese Medicine, Taipei 112, Taiwan; 4 Department of Life Sciences and Institute of Genome Sciences, School of Life Science, National YangMing University, Taipei 112, Taiwan; 5 Institute of Biotechnology, National Cheng Kung University, Tainan 701, Taiwan; 6 Department of Life Sciences, National Cheng Kung University, Tainan 701, Taiwan

**Keywords:** Baeocytous cyanobacterium, Comparative genomics, Cyanobiont, GI1, Myxosarcina, Pleurocapsales, Terpios hoshinota

## Abstract

To date, genome sequences (complete or in draft form) from only six baeocytous cyanobacteria in four genera have been reported: *Xenococcus*, *Chroococcidiopsis*, *Pleurocapsa*, and *Stanieria*. To expand our knowledge on the diversity of baeocytous cyanobacteria, this study sequenced the genome of GI1, which is a *Myxosarcina*-like baeocytous cyanobacterium. GI1 is of interest not only because of its phylogenetic niche, but also because it is a cyanobiont isolated from the marine cyanobacteriosponge *Terpios hoshinota*, which has been shown to cause the death of corals. The ~7 Mb draft GI1 genome contains 6,891 protein-coding genes and 62 RNA genes. A comparison of genomes among the sequenced baeocytous cyanobacterial strains revealed the existence or absence of numerous discrete genes involved in nitrogen metabolism. It will be interesting to determine whether these genes are important for cyanobacterial adaptations and interactions between cyanobionts and their marine sponge hosts.

## Introduction

In the latest (second) edition of *Bergey’s Manual of Systematic Bacteriology*, cyanobacteria are classified into five subsections (“orders”) [1]. All members in Subsection II (order *Pleurocapsales*) reproduce (exclusively or partially) via multiple fission, which produces small reproductive cells called baeocytes [2]; these species are thus described as “baeocytous”. Baeocytous species are further divided into seven genera according to developmental characteristics, such as: the contribution of baeocyte formation to reproduction, the morphology of cell aggregates associated with successive binary fission in vegetative cells, and the presence of fibrous cell walls at the onset of baeocyte formation. The seven genera are *Cyanocystis *, *Dermocarpella *, *Stanieria *, *Xenococcus *, *Chroococcidiopsis *, *Pleurocapsa *, and *Myxosarcina *[2]. The taxa in Subsection II present considerable diversity in terms of physiology and ecology. Most baeocytous species are solitary (free-living) entities, which can be found in water or on land. Intertidal zones show a particularly rich diversity of baeocytous species, most of which are epilithic or endolithic [2]. A number of species associate with lichen [3] or sporadically occur as extracellular symbionts of marine sponges [4]. *Terpios hoshinota* is a marine cyanobacteriosponge that infests coral reefs in west Indo-Pacific regions [5]. *T. hoshinota* infestations have been named “black disease” because these sponges tend to overgrow live corals, resulting in the formation of black encrustations, which can spread within a few days and shut down photosynthesis. This causes the death of the coral, with none of the coral pulps able to regenerate following encrustation. In 2006, an unprecedented outbreak of black disease occurred in the waters of Green Island, located southeast of Taiwan. In that outbreak, more than 30% of coral were overgrown by sponge [6]. Little is known about the nature of coral black disease, although *Montipora aequituberculae* corals appear to be particularly susceptible. *T. hoshinota* is associated with a substantial quantity of cyanobacteria, mainly *Aphanocaps* type, which lives intercellularly within the sponge [5]. The cyanobacteria associated with *T. hoshinota* are able to perform photosynthesis; therefore, it has been suggested that cyanobionts provide nutrients to support the spread of their host [6]. In this work, we succeeded in purifying a cyanobacterium associated with *T. hoshinota* from Green Island, called GI1. We then cultivated the organisms in the laboratory to study their taxonomy and physiology. Specifically, we describe the morphological, biochemical, and genomic properties of GI1, which resemble those of a *Myxosarcina * species [2]. The genome sequence of GI1 may also provide insight into symbiotic interactions between cyanobionts and their marine sponge hosts.

## Organism information

### Classification and features

**Figure 1:**
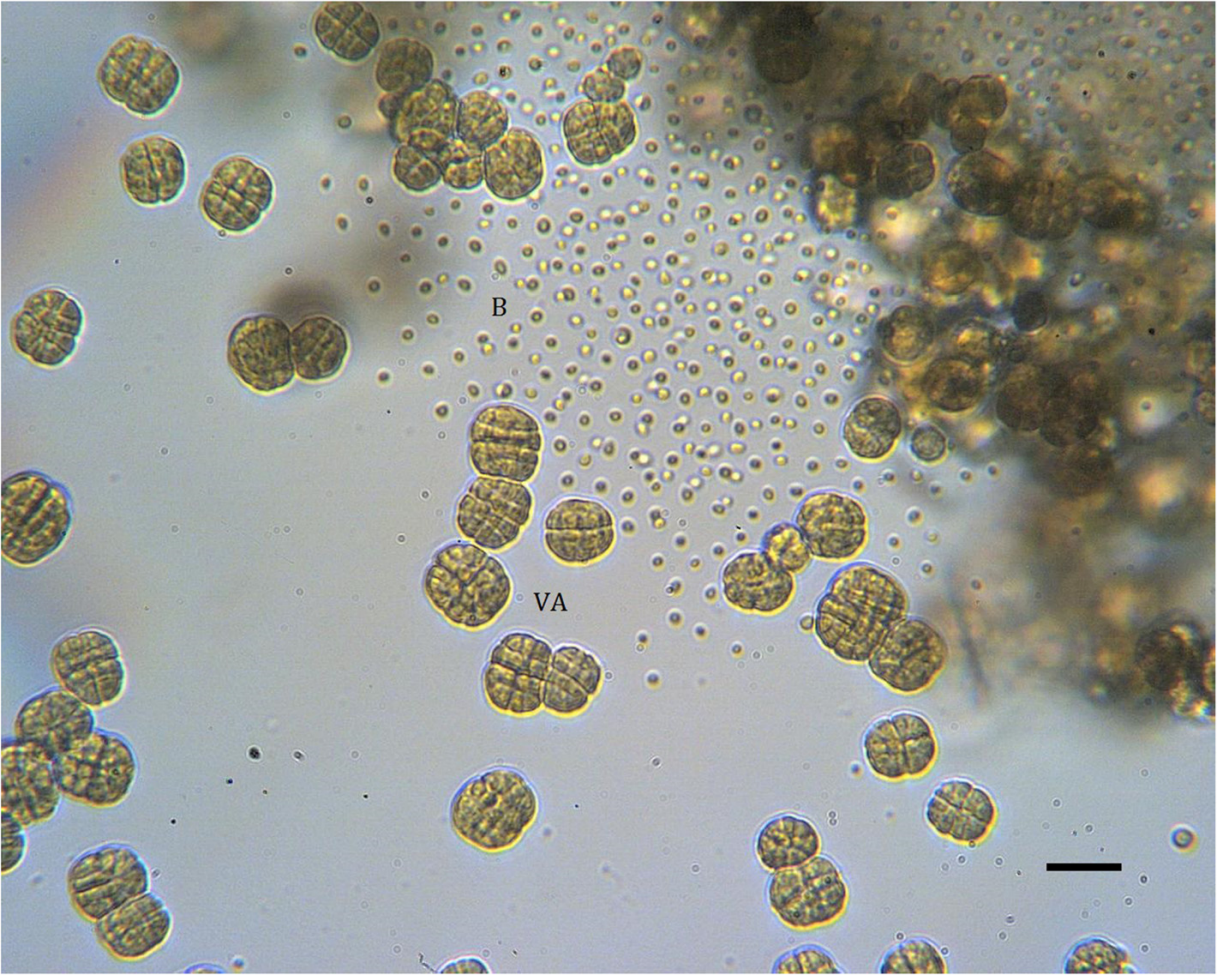
Characteristic vegetative cell aggregates (VA) and baeocytes (B) of GI1 observed under a light microscope. Cells were cultivated in ASN III medium for 1 month (~ late exponential phase). Bar = 20 μm.

**Figure 2:**
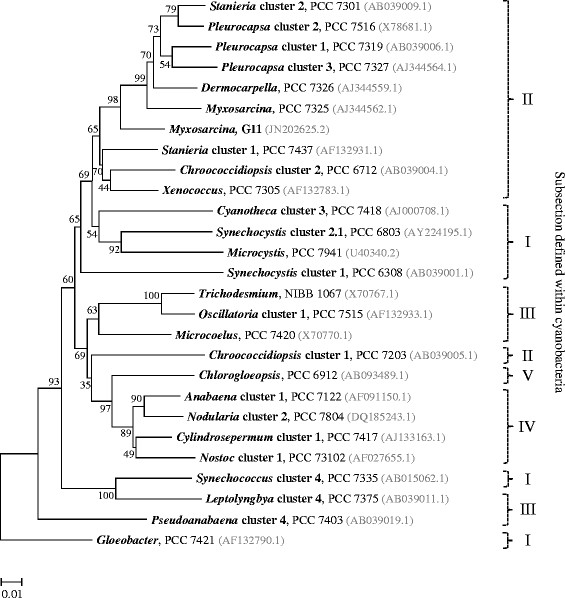
Phylogenetic position of *Myxosarcina* sp. strain GI1 within cyanobacteria. The 16S rRNA gene sequences of GI1 and type strains belonging to different cyanobacterial subsections were subjected to phylogenetic analysis using MEGA5 software [31] in conjunction with the multiple alignment program CLUSTAL W to construct a maximum-likelihood tree, using bootstrap values of 1000 replicates. The GenBank accession numbers for each stain are shown in parenthesis.

A coral sample (*Montipora* sp.) overgrown by *T. hoshinota* was collected from the sub-tidal zone of Green Island in 2007. Black scrapings from the surface of the sample were suspended in sterile seawater and then streaked onto plates prepared by supplementing ASN-III medium [7] with 0.8% agar (ASN-III agar plates). After two months, only one type of cyanobacterium, characterized by a punctiform shape and blackish color, was found on the plate. This cyanobacterium was purified by successively transferring and streaking onto the same type of plates at two month intervals. An axenic culture was then established and added to our collection as strain GI1. This strain produced coccoid and motile baeocytes, which reacted photactically and lost mobility as they enlarged into spherical vegetative cells. Most of the vegetative cells performed successive binary fission in three planes, which resulted in the formation of cubic or irregular cell aggregates and eventually produced baeocytes (Figure 1). Baeocyte diameters (2.3 ± 0.2 μm) differed little from those of parental (mature) vegetative cells (3.7 ± 0.7 μm) that were preparing to release baeocytes. The vegetative cells in GI1 had an average volume only 4.2 times larger than that of a newly released baeocytes; thus, each vegetative cell could produce no more than 4 baeocytes. These characteristics suggest that GI1 belongs to the *Myxosarcina *[2]. Phylogenetic analysis of 16S rRNA gene sequences led to the segregation of Subsection II cyanobacteria into two groups in the tree (Figure 2). The first group contained *Chroococcidiopsis * cluster 1, which is similar to heterocyst-forming cyanobacteria; the second group contained the bulk of Subsection II cyanobacteria, including GI1. Note that with high bootstrap support, GI1 did not form a sister clade with *Myxosarcina *PCC 7325, which was located in the same clade containing *Pleurocapsa *, *Dermocarpella *, and *Stanieria * cluster 2. *Stanieria * cluster 2 also failed to form a sister clade with *Stanieria * cluster 1. These observations suggest that the phylogeny of the 16S rRNA gene sequence is not consistent with the taxonomic relationships among baeocytous cyanobacteria. GI1 is a facultative photoheterotroph. Supplementing the ASN-III medium with yeast extract and glucose in 1 and 2 g/L^−1^ stimulated the growth of GI1 but inhibited the growth of *Myxosarcina * strain PCC 7312, indicating that the ability of GI1 to use organic resources exceeds that of PCC 7312. The classification and general features of *Myxosarcina * sp. strain GI1 are summarized in Table 1.

**Table 1:** **Classification and general features of** ***Myxosarcina*** **sp. strain GI1 according to MIGS recommendations**[8]

**MIGS ID**	**Property**	**Term**	**Evidence code**^**a**^
	Current classification	Domain *Bacteria*	TAS [29]
		Phylum *Cyanobacteria*	TAS [1]
		Order *Pleurocapsales*	TAS [2]
		Genus *Myxosarcina*	TAS [2]
		Species Unknown	
		Type strain PCC 7325	TAS [2]
	Gram stain	Not reported	
	Cell shape	Spherical (baeocyte)	IDA
		Cubic (vegetative cell)	IDA
	Motility	Gliding (newly-born baeocyte)	IDA
	Sporulation	Not reported	
	Temperature range	Not reported	
	Optimum temperature	Not reported	
	pH range; Optimum	Not reported	
	Carbon source	Not reported	
	Energy source	Phototrophic	IDA
MIGS-6	Habitat	Marine	IDA
MIGS-6.3	Salinity	3-4%	IDA
MIGS-22	Oxygen	Aerobic	IDA
MIGS-15	Biotic relationship	Symbiont	IDA
MIGS-14	Pathogenicity	Non-pathogen	IDA
MIGS-4	Geographic location	Green Island, Taiwan	IDA
MIGS-5	Sample collection time	July, 2007	IDA
MIGS-4.1	Latitude	22.6769444	IDA
MIGS-4.2	Longitude	121.4930556	IDA
MIGS-4.3	Depth	10 feet	IDA
MIGS-4.4	Altitude	Not applicable	

^a^Evidence codes - IDA: Inferred from Direct Assay; TAS: Traceable Author Statement (i.e., a direct report exists in the literature); NAS: Non-traceable Author Statement (i.e., not directly observed for the living, isolated sample, but based on a generally accepted property for the species, or anecdotal evidence). These evidence codes are from the Gene Ontology project [30].

## Genome sequencing information

### Genome project history

The project information and its association with MIGS version 2.0 compliance [8] are summarized in Table 2. The genome was first sequenced in 2010 and this work provides a high-quality draft of genome. The assembled contigs have been deposited in NCBI.

**Table 2:** Project information

**MIGS ID**	**Property**	**Term**
MIGS-31	Finishing quality	High quality draft
MIGS-28	Libraries used	1 paired-end and 3 mate-pairs
MIGS-29	Sequencing platforms	Illumina (GAIIx, Hiseq2000)
MIGS-31.2	Fold coverage	1186x
MIGS-30	Assemblers	ALLPATHS-LG (v47833)
MIGS-32	Gene calling method	RAST webserver (Glimmer 3)
	Locus Tag	KV40 (prefix)
	Genbank ID	GI:685984682
	Genbank Date of Release	Sep 14, 2014
	GOLD ID	Gi0078648
	BIOPROJECT	PRJNA259928
MIGS-13	Source Material Identifier	N.A.
	Project relevance	Cyanobacterial ecology, cyanobiont

### Growth conditions and genomic DNA preparation

A single colony of GI1 was selected from the ASN-III agar plate and transferred into a 1 L serum bottle with 200 mL of ASN-III medium. The culture was shaken (90 rpm), aerated (0.2 volume per volume per minute, VVM), and illuminated laterally at 27.0 μmol photons · m^−2^ · s^−1^, as measured at the surface of the bottle. Cells were then cultivated in a 12:12 light–dark cycle until the late exponential phase of growth. The cells in each culture were harvested by centrifugation at 5,000 × g for 15 min, rinsed twice using 10 mL deionized water, and extracted using Tri-Total Nucleic Acid Isolation Reagent (Geneaid, New Taipei City, Taiwan) to obtain genomic DNA. Extraction was performed according to manufacturer guidelines. Genomic DNA of GI1 was quantified using the Quant-iT dsDNA BR Assay Kit (Invitrogen, Carlsbad, CA, USA) and quality checked on 0.6% agarose gel. Twenty micrograms of DNA was sheared using a Bioruptor ultrasonicator (Diagenode, Liège, Belgium) set at power on for 30 sec and power off for 30 sec. The fragmented DNA was then separated using either 1.5% or 0.6% agarose gel electrophoresis to obtain DNA strands of various lengths.

### Genome sequencing and assembly

One paired-end (PE) and three mate-pair (MP) libraries of GI1 genomic DNA were prepared. The PE library (insert size: 143 ± 50 bp) was sequenced on Illumina GAIIx at the Bioresource Research Center, National Cheng Kung University, Taiwan. The three MP libraries (insert sizes of approximately 3, 5, and 9 kb) were sequenced on Illumina HiSeq 2000 at Yourgene Bioscience, Taiwan. Prior to assembly, low quality reads in the PE and 3 kb MP libraries were filtered out. A read was considered low quality if (1) it contained an unknown base “N”, (2) the lowest quality score was less than 30, or (3) more than 95% of the bases were identical. The reads of the 3 kb MP library were further trimmed to a length of 60 bp. The 5 kb and 9 kb MP libraries were prepared using Illumina’s Nextera kit. Reads containing the Nextera adaptor sequence were retained and the adaptor parts were removed. Resulting reads shorter than 40 bp or containing an unknown base “N” were further discarded. The processed reads of all four libraries accounted for an 1186X coverage based on a 7 Mb genome. These reads were assembled using ALLPATHS-LG (v47833) [9],[10] with all parameters set to default.

### Genome annotation

Protein coding genes were predicted using Glimmer 3 [11] and annotated using the RAST webserver [12],[13]. The tRNA genes and rRNA genes were identified using tRNAscanSE (v1.3.1) [14],[15] and RNAmmer (v1.2) [16], respectively. For annotations of COG, Pfam, TIGRfam, and PRK, Conserved Domain Database [17]-[23] was downloaded from NCBI and the predicted proteins were aligned to each dataset using RPSblast (v2.2.29) [24]; all parameters were set to default. For each protein, the best alignment (highest score) was selected for annotation. To facilitate genome comparison, protein and nucleotide sequences of the six baeocytous cyanobacterial strains were obtained from either NCBI FTP site (*Chroococcidiopsis thermalis *PCC 7203, *Pleurocapsa * sp. PCC 7319 and PCC 7327, *Stanieria **cyanosphaera*PCC 7437, and *Xenococcus * sp. PCC 7305) or JGI database (*Chroococcidiopsis * sp. PCC 6712) for annotation as described above.

## Genome properties

**Figure 3:**
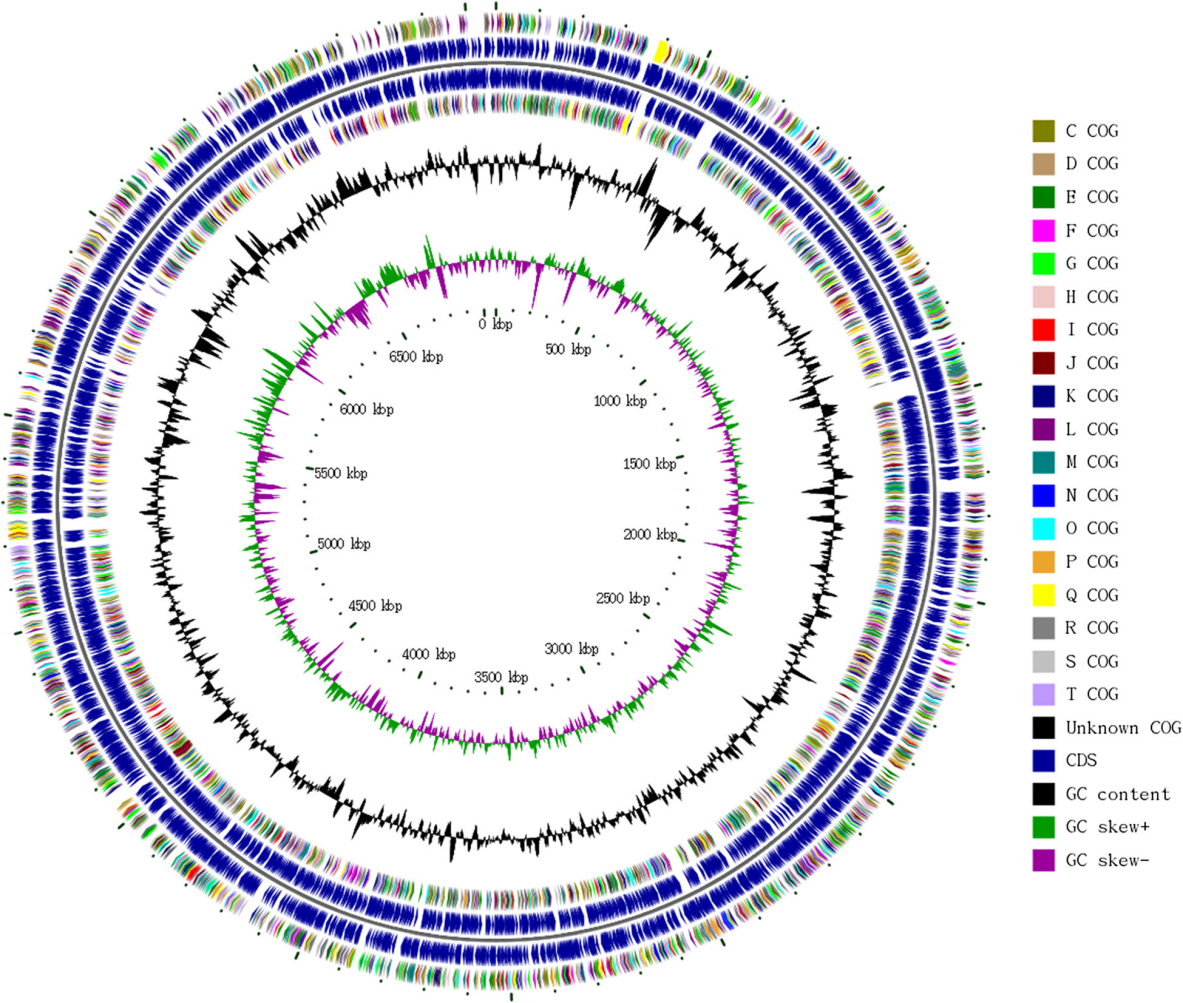
Circular map of GI1 chromosome.

The draft genome of GI1 contained 7.06 M bp in 76 contigs (or 21 scaffolds); the N50 length of the contigs was 195,043 bp (Table 3). The GC content was 40.1%. Gene annotation revealed 6891 protein coding genes, 6 rRNA genes, and 56 tRNA genes. COG annotations of protein coding genes are presented in Table 4. Figure 3 presents the genome atlas of GI1.

**Table 3:** Genome statistics

**Attribute**	**Value**
Genome size (bp)	7,069,859
DNA coding (bp)	5,958,317
DNA G+C (bp)	2,834,956
DNA scaffolds	21
Total genes	6,953
Protein coding genes	62
RNA genes	62
Pseudo genes	
Genes in internal clusters	
Genes with function prediction	
Genes assigned to COGs	4,118
Genes with Pfam domains	4,730
Genes with signal peptides	
Genes with transmembrane helices	
CRISPR repeats	

**Table 4:** Number of genes associated with the 25 general COG functional categories

**Code**	**Value**	**% age**^**a**^	**Description**
J	196	2.84	Translation
A	0	0	RNA processing and modification
K	274	3.98	Transcription
L	307	4.46	Replication, recombination and repair
B	2	0.03	Chromatin structure and dynamics
D	60	0.87	Cell cycle control, mitosis and meiosis
Y	0	0	Nuclear structure
V	74	1.07	Defense mechanisms
T	396	5.75	Signal transduction mechanisms
M	274	3.98	Cell wall/membrane biogenesis
N	41	0.59	Cell motility
Z	0	0	Cytoskeleton
W	0	0	Extracellular structures
U	80	1.16	Intracellular trafficking and secretion
O	193	2.80	Posttranslational modification, protein turnover, chaperones
C	239	3.47	Energy production and conversion
G	212	3.08	Carbohydrate transport and metabolism
E	299	4.34	Amino acid transport and metabolism
F	87	1.26	Nucleotide transport and metabolism
H	194	2.82	Coenzyme transport and metabolism
I	96	1.39	Lipid transport and metabolism
P	287	4.16	Inorganic ion transport and metabolism
Q	151	2.19	Secondary metabolites biosynthesis, transport and catabolism
R	677	9.82	General function prediction only
S	401	5.82	Function unknown
-	2773	40.24	Not in COGs

^a^The total is based on the total number of protein coding genes in the genome.

## Insights from the genome sequence

The seven baeocytous cyanobacterial genomes (including GI1) are compared in Table 5. By comparing COG annotations, we identified 13 genes that existed in all baeocytous cyanobacteria except GI1 (Table 6) and 36 genes that only appeared in GI1 (Table 7). Many products of these genes (e.g. UreE, SpeA, and GltD in Table 6 and ArgR, COG2070, HutG, COG4262, and NtrB in Table 7) are related to nitrogen metabolism. It can therefore be surmised that these genes participate in nitrogen cycles between cyanobionts and their hosts. Moreover, many GI1-specific genes are involved in processing a wide range of organic compounds as carbon, nitrogen, or energy sources. The putative products encoded by these genes include COG 2070 (dioxygenases related to 2-nitropropane dioxygenase) [25], HutG (N-formylglutamate amidohydrolase) [26], CelA (cellobiohydrolase A) [27], and Hdrc (heterodisulfide reductase, subunit C) [28]. These enzymes are rarely found in cyanobacteria but are common among heterotrophic bacteria and fungi. Exploring the origins and functions of these genes in GI1 will no doubt produce interesting results.

**Table 5:** Genome statistics comparison among baeocytous cyanobacteria

**Genome name**	***Chroococcidiopsis***			***Pleurocapsa***	***Stanieria***	***Xenococcus***	***Myxosarcina***
	**PCC 7203**	**PCC 6712**	**PCC 7327**	**PCC 7319**	**PCC 7437**	**PCC 7305**	**GI1**
Genome size (bp)	6,689,401	5,720,887	4,986,817	7,386,997	5,544,990	5,929,641	7,069,859
G + C content (%)	44.5	35.3	45.2	38.7	36.2	39.7	40.1
Total genes	6,033	5,176	4,665	5,896	5,041	5,419	6,953
Protein-coding genes	5,752	5,116	4,268	5,762	4,781	5,373	6,891
Protein with function prediction	3,728	3,988	2,848	1,256	3,393	3,694	3,664
RNA genes	58	60	56	52	52	46	62
COGs	3,980	3,372	2,970	3,896	3,207	3,458	4,118
%COGs	65.97%	65.15%	63.67%	66.08%	63.62%	63.81%	59.23%
Pfam	4,530	3,918	3,421	4,495	3,716	4,049	4,730
%Pfam	75.09%	75.70%	73.33%	76.24%	73.72%	74.72%	68.03%
TIGRfam	3,107	2,527	2,361	2,872	2,489	2,584	3,078
%TIGRfam	51.50%	48.82%	50.61%	48.71%	49.38%	47.68%	44.27%
SMART	1,338	1,202	1,025	1,294	1,151	1,154	1,452
%SMART	22.18%	23.22%	21.97%	21.95%	22.83%	21.30%	20.88%
PRK	3,211	2,603	2,484	2,968	2,549	2,629	3,154
%PRK	53.22%	50.29%	53.25%	50.34%	50.57%	48.51%	45.36%

**Table 6:** Putative gene products (obtained from genome wide COG assignment) existing in all sequenced baeocytous cyanobacteria except GI1

**Symbol**	COG code^*^	**Description**
UreE	O	Urease accessory protein UreE
SpeA	E	Arginine decarboxylase (spermidine biosynthesis)
GltD	E, R	NADPH-dependent glutamate synthase beta chain
COG5551	V	CRISPR system related protein, RAMP
COG2378	K	Predicted transcriptional regulator
COG4235	O	Cytochrome c biogenesis factor
MnhB	P	Multisubunit Na^+^/H^+^ antiporter, MnhB subunit
COG4942	D	Membrane-bound metallopeptidase
ERG3	I	Sterol desaturase
COG1468	V	CRISPR-associated protein Cas4 (RecB family)
COG1343	V	CRISPR-associated protein Cas2
COG2607	R	Predicted ATPase (AAA+ superfamily)
COG3689	S	Predicted membrane protein

^*^D: cell division and chromosome partitioning; E: amino acid transport and metabolism; I: lipid metabolism; K: transcription; O: posttranslational modification, protein turnover, chaperones; P: inorganic ion transport and metabolism; Q: secondary metabolites biosynthesis, transport, and catabolism; R: general function prediction only; S: function unknown; V: defense mechanisms.

**Table 7:** Putative gene products (obtained from genome wide COG assignment) that only appeared in GI1

**Symbol**	COG code^*^	**Description**
ArgR	K	Arginine repressor
COG2070	R	Dioxygenases related to 2-nitropropane dioxygenase
HutG	E	N-formylglutamate amidohydrolase
COG4262	R	Predicted spermidine synthase with an N-terminal membrane domain
NtrB	T	Signal transduction histidine kinase, nitrogen specific
IQG1	D, T	Protein involved in regulation of cellular morphogenesis/cytokinesis
COG3635	G	Predicted phosphoglycerate mutase, AP superfamily
Rof	K	Transcriptional antiterminator
COG4092	M	Predicted glycosyltransferase involved in capsule biosynthesis
PRI2	L	Eukaryotic-type DNA primase, large subunit
ERG12	I	Mevalonate kinase
MazG	R	Predicted pyrophosphatase
CelA	G	Cellobiohydrolase A (1,4-beta-cellobiosidase A)
COG4101	G	Predicted mannose-6-phosphate isomerase
COG1107	L	Archaea-specific RecJ-like exonuclease
COG4129	S	Predicted membrane protein
PepD	E	Dipeptidase
COG4849	R	Predicted nucleotidyltransferase
COG3103	T	SH3 domain protein
AbiF	V	Abortive infection bacteriophage resistance protein
DRG	R	Predicted GTPase
COG4186	R	Predicted phosphoesterase or phosphohydrolase
COG3292	T	Predicted periplasmic ligand-binding sensor domain
COG4227	L	Antirestriction protein
COG2837	P	Predicted iron-dependent peroxidase
COG4109	K	Predicted transcriptional regulator containing CBS domains
MecR1	K, T	Antirepressor regulating drug resistance
Gcd	G	Glucose dehydrogenase
COG3588	G	Fructose-1,6-bisphosphate aldolase
COG1289	S	Predicted membrane protein
COG4341	R	Predicted HD phosphohydrolase
SRP1	U	Karyopherin (importin) alpha
COG3886	L	Predicted HKD family nuclease
COG1444	R	Predicted P-loop ATPase fused to an acetyltransferase
COG1204	R	Superfamily II helicase
HdrC	C	Heterodisulfide reductase, subunit C

^*^C: energy production and conversion; D: cell division and chromosome partitioning; E: amino acid transport and metabolism; G: carbohydrate transport and metabolism; I: lipid metabolism; K: transcription; L: DNA replication, recombination, and repair; M: cell envelope biogenesis, outer membrane; P: inorganic ion transport and metabolism; R: general function prediction only; S: function unknown; T: signal transduction mechanisms; U: intracellular trafficking and secretion; V: defense mechanisms.

## Conclusions

The assembly and analysis of GI1 genome revealed distinctive genes involved in nitrogen metabolism and utilization of a large array of organic compounds. The GI1 genome is thus valuable for studying interactions between GI1 and its marine sponge host.

## Abbreviations

MP: Mate-pair

PE: Paired-end

VVM: Volume per volume per minute

## Competing interests

The authors declare that they have no competing interests.

## Authors' contributions

YC and TL designed and carried out the experiments. CY, CL, HS, TC, and CH performed the data analysis and drafted the manuscript. All authors read and approved the final manuscript.
